# G2GSnake: a Snakemake workflow for host–pathogen genomic association studies

**DOI:** 10.1093/bioadv/vbad142

**Published:** 2023-10-04

**Authors:** Zhi Ming Xu, Olivier Naret, Mariam Ait Oumelloul, Jacques Fellay

**Affiliations:** School of Life Sciences, École Polytechnique Fédérale de Lausanne, Lausanne 1015, Switzerland; Swiss Institute of Bioinformatics, Lausanne 1015, Switzerland; School of Life Sciences, École Polytechnique Fédérale de Lausanne, Lausanne 1015, Switzerland; Swiss Institute of Bioinformatics, Lausanne 1015, Switzerland; School of Life Sciences, École Polytechnique Fédérale de Lausanne, Lausanne 1015, Switzerland; Swiss Institute of Bioinformatics, Lausanne 1015, Switzerland; School of Life Sciences, École Polytechnique Fédérale de Lausanne, Lausanne 1015, Switzerland; Swiss Institute of Bioinformatics, Lausanne 1015, Switzerland; Precision Medicine Unit, Lausanne University Hospital and University of Lausanne, Lausanne 1011, Switzerland

## Abstract

**Summary:**

Joint analyses of paired host and pathogen genome sequences have the potential to enhance our understanding of host–pathogen interactions. A systematic approach to conduct such a joint analysis is through a “genome-to-genome” (G2G) association study, which involves testing for associations between all host and pathogen genetic variants. Significant associations reveal host genetic factors that might drive pathogen variation, highlighting biological mechanisms likely to be involved in host control and pathogen escape. Here, we present a Snakemake workflow that allows researchers to conduct G2G studies in a reproducible and scalable manner. In addition, we have developed an intuitive R Shiny application that generates custom summaries of the results, enabling users to derive relevant insights.

**Availability and implementation:**

G2GSnake is freely available at: https://github.com/zmx21/G2GSnake under the MIT license.

## 1 Introduction

Hosts and pathogens are involved in an evolutionary battle that involve successive rounds of evolution from both sides (Daugherty and Malik 2012). Pathogens constantly evolve to escape from host immunity or other control mechanisms, while simultaneously hosts are also under evolutionary pressure from pathogens. The signatures of this evolutionary battle are reflected on both genomes. Therefore, joint analyses of host and pathogen genomes offer an opportunity to re-capitulate such processes and to identify specific genetic loci involved in host–pathogen interactions.

An effective method to jointly analyze paired host and pathogen genomes is through the “genome-to-genome” (G2G) approach. This hypothesis-free approach involves searching for significant associations between all pairs host and pathogen variants (Fellay and Pedergnana 2020), reflecting either host selection pressure on the pathogen or pathogen selection pressure on the host. An example where such evolutionary conflict often occurs is between host genetic loci that are involved in pathogen control and pathogen genetic loci that are involved in immune escape. Indeed, G2G studies conducted in viruses such as human immunodeficiency virus (HIV) (Bartha *et al.* 2013) and hepatitis C virus (HCV) (Ansari *et al.* 2017) have highlighted relevant viral immune evasion mechanisms. However, G2G studies conducted in pathogens such as Epstein–Barr virus (EBV) (Rüeger *et al.* 2021), *Mycobacterium tuberculosis* (Phelan *et al.* 2023), and *Plasmodium falciparum* (Band *et al.* 2022) illustrates the potential of the approach to also uncover other important host–pathogen interaction mechanisms.

To our knowledge, existing software used for G2G studies are mostly limited to specific pathogens, and lacks generalizable computational or result visualization capabilities. Given that the G2G approach could be systematically applied to many other pathogens, we present here a Snakemake workflow that can be generalized to any paired host–pathogen genomic datasets. We have also developed an interactive R Shiny app that enables users to generate custom queries and visualizations of the results.

## 2 Methods

### 2.1 Workflow

**Figure 1.**
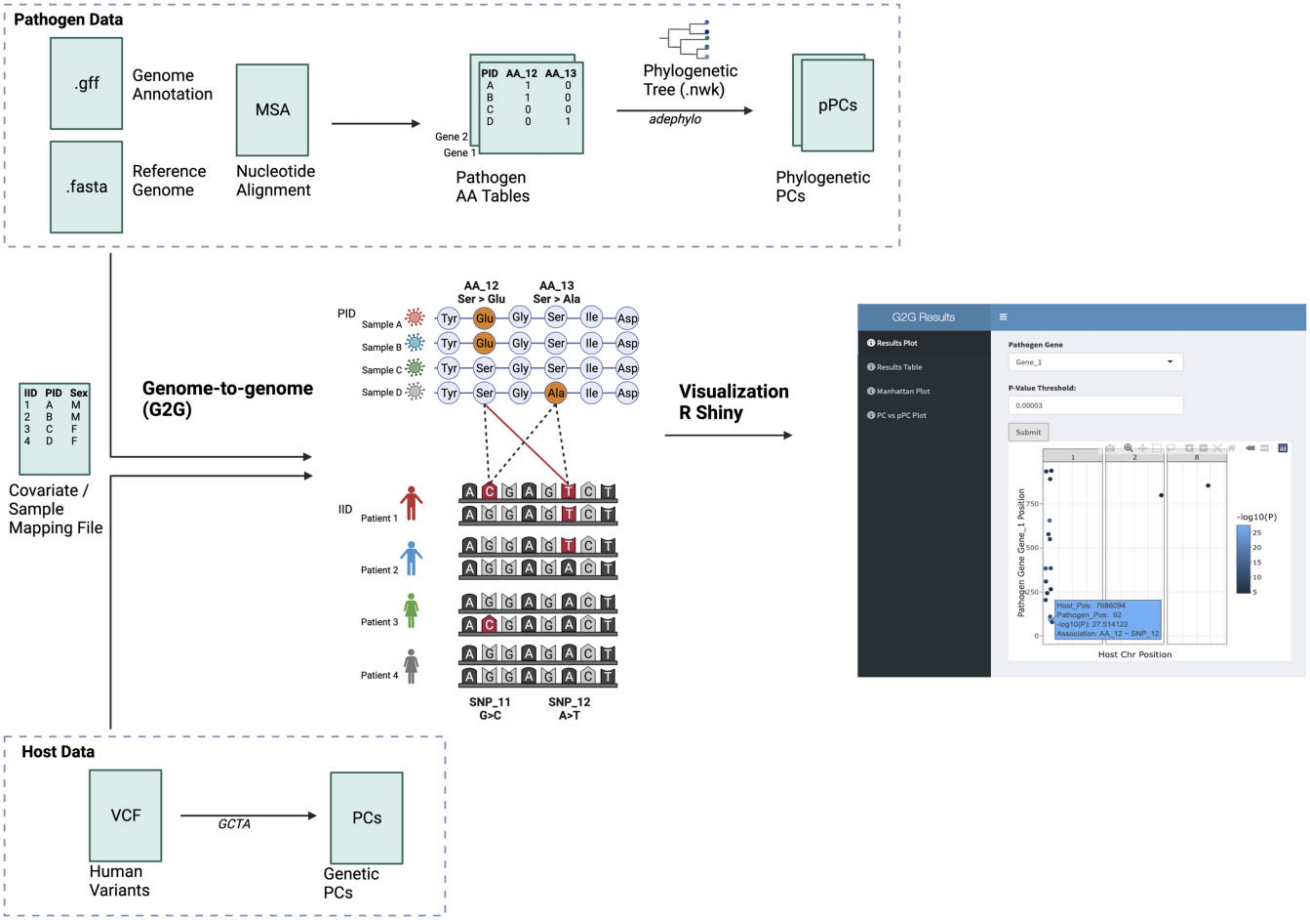
Summary of G2Gsnake. The workflow relies on three main input sources: (i) pathogen genetic data, (ii) host genetic data, and (iii) a sample mapping file along with additional covariates. Associations between all pathogen and host genetic variants are then tested under the genome-to-genome (G2G) framework. Pathogen and host genetic principal components are included as covariates to correct for stratification. Finally, results can be visualized in the R Shiny app. Created with BioRender.com.

A summary of the functionalities of the G2GSnake software is shown in Fig. 1. In brief, the workflow relies on: (i) pathogen genetic data, either a nucleotide multiple sequence alignment or an amino acid matrix for each gene, (ii) host genetic data, in the form of a VCF file, and (iii) a sample mapping file that lists sample pairs and additional covariates. Genetic principal components are calculated for both the host and pathogen to correct for stratification. Associations between all host and pathogen variants are then tested under the G2G framework, corrected for principal components and any provided covariates. A directed acyclic graph for a typical workflow is shown in Supplementary Fig. S1. All jobs are executed within a provided docker container to ensure reproducibility and cross-platform compatibility. References to all external tools used in the pipeline can be found in supplementary materials.

### 2.2 Pathogen genetic data

On the pathogen side, a binary matrix is required for each gene to analyze, encoding the presence or absence of amino-acid variants in each pathogen sample. Variants should be separated by gene, and multi-allelic variants should be decomposed and encoded as separate variants. We also provide functionalities that derive such a matrix from a multiple-sequence nucleotide alignment file (fasta) using custom R scripts and nextalign (Aksamentov *et al.* 2021) for translation. In such an instance, a reference genome (fasta) and annotation (gff) file is also required to extract nonsynonymous variants. Multi-allelic variants would be automatically decomposed. Next, given that a phylogenetic tree in newick format is provided, phylogenetic principal components are calculated using the adephylo package in R (Jombart *et al.* 2010). As a general approach, we recommend that a phylogenetic tree be built from genes concatenated per individual, given that variability within a gene may be limited for slower-evolving pathogens. Alternatively, standard principal components can be calculated. Finally, pathogen variants are filtered based on user-specified allele frequency and missingness thresholds (fraction of samples with null calls at a given variant site).

### 2.3 Host genetic data

On the host side, a VCF file is required. Single-nucleotide polymorphisms, insertion–deletions (indels), and structural variants are all supported. However, multi-allelic variants should be decomposed and indels should be normalized beforehand. Quality control procedures are based on user-defined thresholds and includes filtering based on missingness, minor allele frequency, and deviation from Hardy–Weinberg equilibrium. Principal components are then calculated using GCTA (Yang *et al.* 2011).

### 2.4 Genome-to-genome study

The software relies on existing tools developed for genome-wide association studies (GWAS) to conduct G2G studies. A case-control GWAS is run for each pathogen variant, treating it as an outcome. Specifically, a regression model is constructed for each pathogen variant *j* and human variant *i*:


$$y_{j}∼\beta_{ij}G_{i}+\sum_{k}\alpha_{k}X_{k}$$

where $y_{j}$ represents a vector (derived from the binary matrix) encoding the presence of pathogen variant *j* (0 or 1), $G_{i}$ represents the genotype dosage vector (derived from the VCF file) of host variant *i*, and $X_{k}$ represents the covariate vector for covariate *k*. Covariates include host and pathogen principal components along with other user-specified covariates.

Users can choose to use PLINK (Chang *et al.* 2015) or REGENIE (Mbatchou et al*.* 2021) to conduct association tests. PLINK was chosen due to its high computational efficiency, while REGENIE was chosen due to its ability to handle rare pathogen variants, which give rise to unbalanced case–control ratios, through the SPA test. While REGENIE is more computationally demanding, both methods can be reasonably applied to a dataset with a size comparable to real-world datasets of past genome-to-genome studies (Supplementary Table S1).

For both methods, un-adjusted *P*-values without correction for multiple testing are reported. To accurately interpret the results, the user should select relevant *P*-value thresholds that accounts for multiple-testing, e.g. by applying Bonferonni correction.

## 3 Results

### 3.1 Simulation studies

To test the validity of our software, we generated four simulated host–pathogen genetic datasets based on the framework described by Naret et al. (2018). Each simulation included 1 000 000 host variants, 500 pathogen variants, and 1000 samples. In simulation A and simulation B, no true associations between host and pathogen variants existed. In simulation A, all host and pathogen variants were not population stratified, while in simulation B, a portion of host and pathogen variants were population stratified (10% and 20%, respectively). In simulation C and simulation D, true associations between host and pathogen variants were created. In simulation C, associations only existed between variants that were not population stratified, while in simulation D, associations also existed between population stratified variants. For all four simulations, the number of host and pathogen variants that belonged to each category is shown in Table 1.

**Table 1.** Number of host and pathogen variants in each category for the four simulation scenarios.^a^

Simulation	Unstratified	Stratified	Associated	Associated and stratified
Pathogen variants				
A	500	0	0	0
B	400	100	0	0
C	200	100	200	0
D	200	100	100	100
Host variants				
A	1 000 000	0	0	0
B	900 000	100 000	0	0
C	899 800	100 000	200	0
D	899 800	100 000	100	100

a All simulations included 1 000 000 host variants, 500 pathogen variants, and 1000 samples.

**Figure 2.**
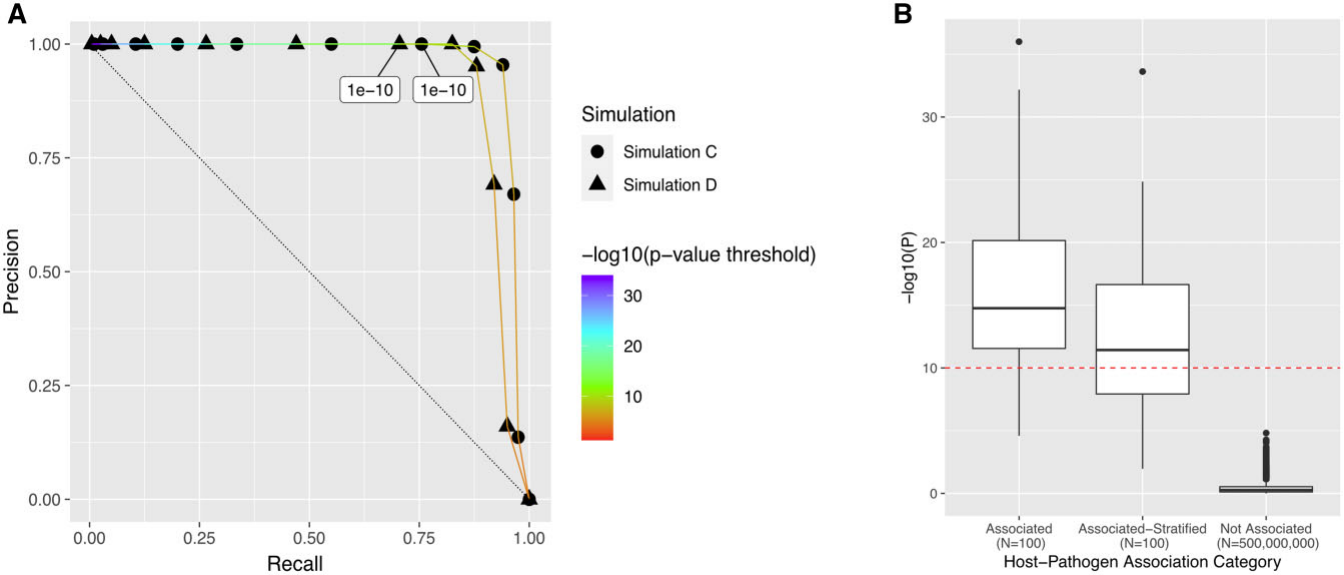
Performance of G2GSnake based on simulation studies. (A) Precision–recall curve when the REGENIE approach was applied to simulation C (no stratified-associated variants) and simulation D (with stratified-associated variants), illustrating the precision–recall trade-off of various *P*-value thresholds. The threshold based on Bonferroni correction (1e−10) is labeled. (B) Association *P*-values based on simulation D for host–pathogen variant pairs that are either: (i) associated, (ii) associated and stratified, or (iii) not associated. The number of variants that belong to each category are indicated in brackets. The dashed line indicates *P*-value threshold based on Bonferroni correction (1e−10).

We next applied G2GSnake to the four simulations using the REGENIE approach. Due to the co-occurrence of pathogen lineages and certain human ancestral groups, spurious associations due to population stratification can occur. To correct for this, we included the top five host and pathogen principal components as covariates. Figure 2A summarizes the precision and recall for simulations with true positives (simulation C and D) based on different *P*-value thresholds. As expected, a higher area under the precision–recall curve (AUPRC) was achieved for simulation C (AUPRC = 0.95) compared to simulation D (AUPRC = 0.91), since simulation D contained associated variants that were also stratified.

Based on the Bonferroni corrected *P*-value threshold (*P*<1×10^−10^, corrected for the number of host–pathogen variant pairs tested under α=0.05), a false positive rate of 0 was achieved for all four simulations (Supplementary Table S2). For simulation C and D, a precision of 1 was achieved for both. A slightly higher recall was achieved for simulation C (0.755) compared to simulation D (0.705). For both simulation C and simulation D, precision was slightly favored over recall due to the relatively conservative nature of Bonferroni correction (Fig. 2A).

Finally, based on simulation D, Fig. 2B compares the *P*-value of associations between variant pairs in different categories. As expected, associations between true G2G associated pairs that were not stratified were detected by the software as strongly associated. For true G2G associated pairs that were stratified, the detected associations were less significant given that principal components cannot fully separate true signal from population stratification. For all other pairs that were not associated, the detected associations were negligible. Few nonassociated pairs were detected as weakly associated likely due to residual stratification that principal components were not able to capture.

### 3.2 Visualization—R Shiny App

To visualize and summarize results from the G2G study, we developed a R Shiny app. The app is launched from a Docker container to ensure cross-platform compatibility. Supplementary Figure S2 illustrates the main functionalities of the app based on results from simulation D, including: (i) a results table which displays summary statistics of all G2G associations below a specified *P*-value threshold (Supplementary Fig. S2A), (ii) a results plot which displays all G2G associations in the specified pathogen gene and below a specified *P*-value threshold (Supplementary Fig. S2B), (iii) a Manhattan or QQ plot for a specified pathogen variant (Supplementary Fig. S2C), and (iv) a correlation plot between host and pathogen principal components (Supplementary Fig. S2D).

## 4 Conclusion

We introduce a scalable and reproducible Snakemake workflow that allows researchers to jointly analyze paired host–pathogen genetic data under the G2G framework. In our implementation, only nonsynonymous pathogen variants were considered as they are more likely to be functionally relevant. However, a limitation is that synonymous variants both within or outside of coding regions that could also have functional consequences, such as variants that affect RNA structure or those within transcription factor binding sites, would be excluded. In addition, variants within diverse accessory loci that are part of the variation of many bacterial species would not conform to multiple-sequence alignments and existing reference genome annotations. To rectify this, users would have to leverage external tools and provide an amino acid matrix for each gene from the pangenome.

In this study, we generated simulations that included 500 independent pathogen variants. This is similar in orders of magnitude to the number of variants tested in previous viral genome-to-genome studies (i.e. in the order of $10^{2}$ to $10^{3}$ variants), but other microbial populations such as bacteria could present more variants (i.e. in the order of $10^{4}$ to $10^{5}$) (Power *et al.* 2017). Analyzing such datasets would be computationally demanding, but still feasible (i.e. run-time of approximately a few days based on the PLINK approach and 110 CPU cores).

For clonal microbial populations, such as *M.tuberculosis*, a large proportion of variants would be highly correlated. In such an instance, the user may want to apply variant pruning to remove highly correlated variants. Furthermore, Bonferroni correction would also be overly conservative. The user may want to utilize other multiple-hypothesis correction procedures, e.g. those based on permutation.

Compared to the software implementation presented by Naret et al. (2018), which was designed primarily as a framework to conduct simulation studies, G2GSnake leverages recent GWAS tools that are more computationally efficient. Furthermore, G2GSnake offers ease-of-use and generalizability, enabling G2G studies to be systematically conducted and interpreted for novel pathogens of interest. Finally, cross-platform compatibility is also guaranteed through docker containers that contain the workflow itself and the R Shiny app.

## Supplementary Material

vbad142_Supplementary_Data
