# Supplementary material for: G2GSnake: a Snakemake workflow for host–pathogen genomic association studies
Source: Bioinform Adv. 2023 Oct 4;3(1):vbad142. doi: 10.1093/bioadv/vbad142 (PMC10576169; doi:10.1093/bioadv/vbad142)
Supplement: vbad142_Supplementary_Data [file vbad142_supplementary_data.pdf]

Supplementary Materials - G2GSnake: A Snakemake workflow for host-pathogen genomic association studies

July 21, 2023

Supplementary Figures

Figure S1: **Directed acyclic graph of snakemake rules** The workflow steps can be summarized as: 1) QC on host genetic data 2) Calculation of host PCA 3) Calculation of pathogen PCA for each pathogen gene 4) Variant frequency filter on pathogen data, separately for each gene 5) Genome-to-Genome study for each gene 6) Write out summary statistics and results files that be read by the R Shiny app

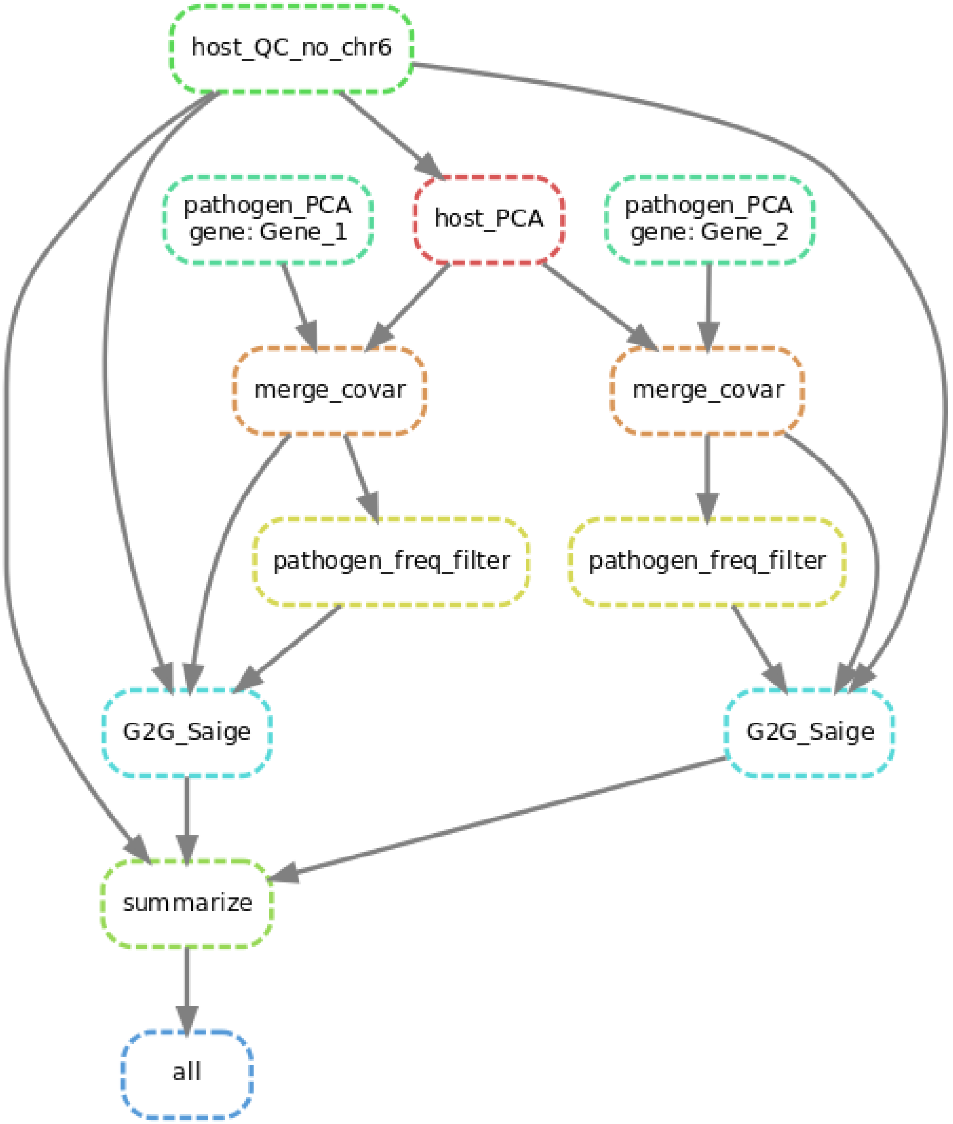

Figure S2: **Functionalities of the R Shiny App, using simulation D as an example. a)** Results table that lists the summary statistics of all associations with p-value below the user-defined threshold **b)** A results plot that displays all associations for a pathogen gene with p-value below the user-defined threshold. X-axis shows the position of the host variant, and y-axis shows the position of the pathogen variant, and color represents the p-value. The pointer is hovering over the top association (between SNP_117 and AA_117). **C)** Manhattan or QQ plot for a specified pathogen variant. The pointer is hovering over the top association (AA_117). Red line indicates Bonferroni corrected significance threshold based on the number of host and pathogen variants, and blue line indicate genome-wide significance threshold based on the number of host variants. **D)** A correlation plot between host and pathogen principal components.

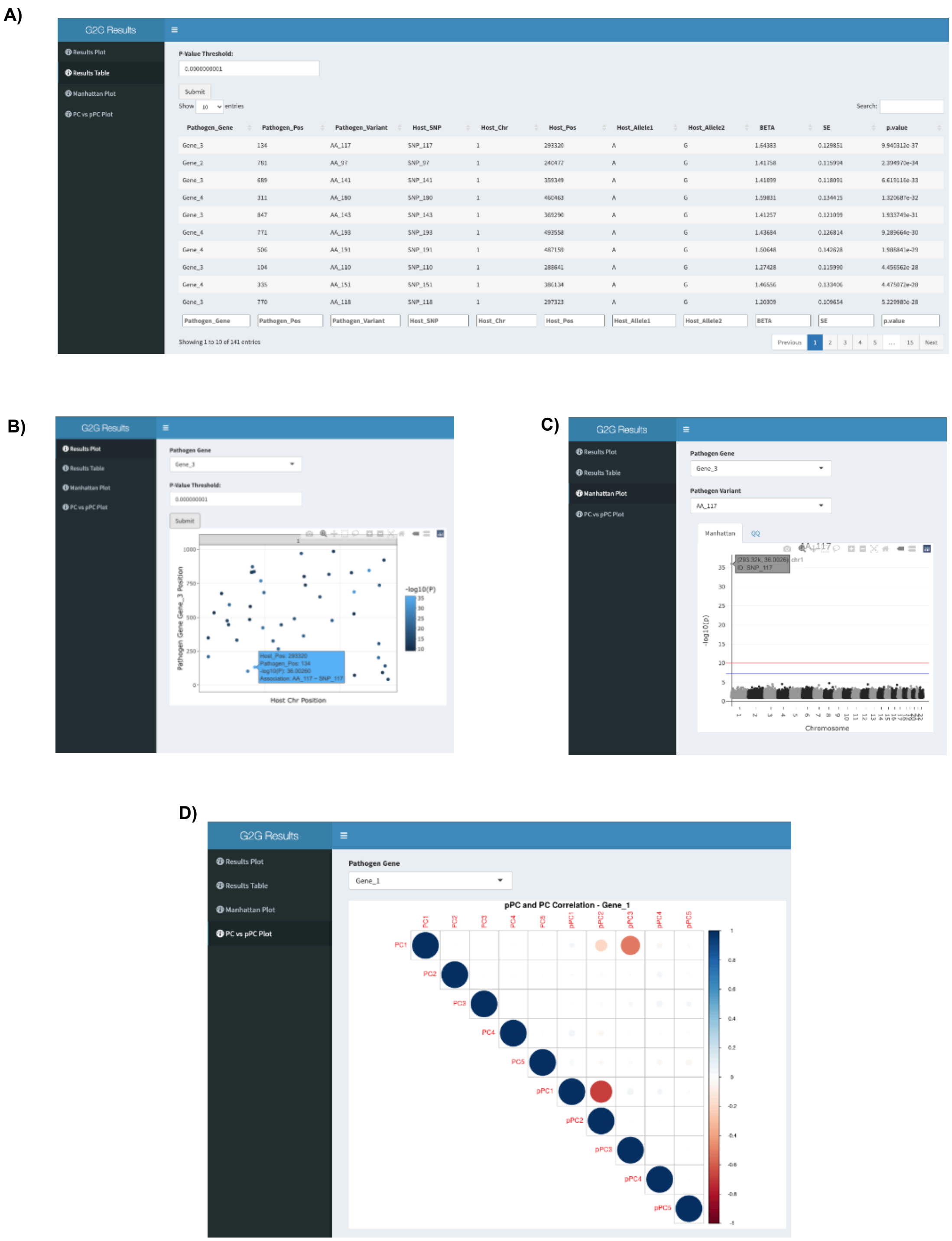

Supplementary Tables

Table S1: Computational performance of G2GSnake based on a dataset with 1000 samples, 1 million host variants, and 500 pathogen variants. Runtime estimates based on 110 CPU cores (AMD Ryzen Threadripper 3990X processors), 252 GB of memory (Corsair CMK64GX4M2D3000C16) and 8TB hard drive (WDC WD8004FRYZ-0)

**Software**	**Runtime (hrs)**	**Number of CPU Cores**	**CPU Hours**
PLINK	0.27	110	26.7
REGENIE (Step 1)	3.40	10	32.0
REGENIE (Step 2)	0.77	110	29.2

Table S2: Performance metrics of G2GSnake for each simulation scenario, based on a p-value threshold derived using Bonferroni correction ($p < 1 \times 10^{-10}$).

**Simulation**	**True Positive Rate (Recall)**	**False Positive Rate**	**Precision**
A	N/A	0	N/A
B	N/A	0	N/A
C	0.755	0	1
D	0.705	0	1

External tools used in G2GSnake

The following external tools were used in G2GSnake:

Pathogen Genetic Data

1. nextalign within the nextstrain package [Aksamentov et al., 2021] for translating nucleotide to amino acid sequences.

2. adephylo package in R [Jombart et al., 2010] for constructing phylogenetic trees.

Host Genetic Data

1. PLINK2 [Chang et al., 2015], for quality control and pre-processing of VCF files.

2. GCTA [Yang et al., 2011], to calculate host principal components

Genome-to-genome study

1. PLINK2 [Chang et al., 2015]

2. REGENIE [Mbatchou et al., 2021]

References

I. Aksamentov et al. Nextclade: clade assignment, mutation calling and quality control for viral genomes. *Journal of Open Source Software*, 6(67):3773, Nov. 2021. ISSN 2475-9066. doi: 10.21105/joss.03773. URL https://joss.theoj.org/papers/10.21105/joss.03773.

C. C. Chang et al. Second-generation PLINK: rising to the challenge of larger and richer datasets. *GigaScience*, 4(1):7, Dec. 2015. ISSN 2047-217X. doi: 10.1186/s13742-015-0047-8. URL https://academic.oup.com/gigascience/article-lookup/doi/10.1186/s13742-015-0047-8.

T. Jombart et al. *adephylo* : new tools for investigating the phylogenetic signal in biological traits. *Bioinformatics*, 26(15):1907–1909, Aug. 2010. ISSN 1367-4811, 1367-4803. doi: 10.1093/bioinformatics/btq292. URL https://academic.oup.com/bioinformatics/article/26/15/1907/188748.

J. Mbatchou et al. Computationally efficient whole-genome regression for quantitative and binary traits. *Nature Genetics*, 53(7):1097–1103, July 2021. ISSN 1061-4036, 1546-1718. doi: 10.1038/s41588-021-00870-7. URL https://www.nature.com/articles/s41588-021-00870-7.

J. Yang et al. GCTA: A Tool for Genome-wide Complex Trait Analysis. *The American Journal of Human Genetics*, 88(1):76–82, Jan. 2011. ISSN 00029297. doi: 10.1016/j.ajhg.2010.11.011. URL https://linkinghub.elsevier.com/retrieve/pii/S0002929710005987.
